# Wide and deep learning based approaches for classification of Alzheimer’s disease using genome-wide association studies

**DOI:** 10.1371/journal.pone.0283712

**Published:** 2023-05-01

**Authors:** Abbas Saad Alatrany, Wasiq Khan, Abir Hussain, Dhiya Al-Jumeily, for the Alzheimer’s Disease Neuroimaging Initiative

**Affiliations:** 1 School of Computer Science and Mathematics, Liverpool John Moores University, Liverpool, United Kingdom; 2 University of Information Technology and Communications, Baghdad, Iraq; 3 Imam Ja’afar Al-Sadiq University, Baghdad, Iraq; 4 Department of Electrical Engineering, University of Sharjah, Sharjah, UAE; Menoufia University, EGYPT

## Abstract

The increasing incidence of Alzheimer’s disease (AD) has been leading towards a significant growth in socioeconomic challenges. A reliable prediction of AD might be useful to mitigate or at-least slow down its progression for which, identification of the factors affecting the AD and its accurate diagnoses, are vital. In this study, we use Genome-Wide Association Studies (GWAS) dataset which comprises significant genetic markers of complex diseases. The original dataset contains large number of attributes (620901) for which we propose a hybrid feature selection approach based on association test, principal component analysis, and the Boruta algorithm, to identify the most promising predictors of AD. The selected features are then forwarded to a wide and deep neural network models to classify the AD cases and healthy controls. The experimental outcomes indicate that our approach outperformed the existing methods when evaluated on standard dataset, producing an accuracy and f1-score of 99%. The outcomes from this study are impactful particularly, the identified features comprising AD-associated genes and a reliable classification model that might be useful for other chronic diseases.

## Introduction

Alzheimer’s disease (AD) is the most prevalent kind of dementia, accounting for 60–70% cases of dementia [1]. It impairs memory, thinking, conduct, and overall capacity to do everyday tasks such as eating and bathing etc. The illness can generally be classified into two subcategories: early-onset Alzheimer’s disease (EOAD) and late-onset Alzheimer’s disease (LOAD) [2]. The EOAD is almost entirely a genetic disease with heritability ranging from 92% to 100% [3] where the affected first-degree relatives account for 35% to 60% of EOAD patients. Usually, the EOAD patients experience their first symptoms between 30 and 65 years of age, with the majority of EOAD patients diagnosed between the ages of 45 and 60 years [4]. In contrast to EOAD, the LOAD affects elderly people (usually over 65 years of age) and has a 90–95% occurring of the AD in overall cases [5]. LOAD appears to be a more complicated illness induced by genetic as well as the environmental factors. For instance, Genome wide associations study (GWAS) of AD reported 44 single-nucleotide polymorphisms (SNP) associated with the LOAD [6]. Likewise, Apolipoprotein E (APOE e4) has been commonly Identified as a risk factor to LOAD [7]. While these works identify some important factors associated with the LOAD, the genetic architecture of the disease and its prediction remains a mystery. Due to lacking cure for AD, identifying the specific genes that are mainly involved in the illness’s progression, will help physicians for the early diagnosis of disease, and therefore will help in monitoring and prevention of the disease.

Recently, variety of computational strategies have been proposed for improving the diagnosis or identification of novel gene candidates associated to AD. For instance, GWAS investigations [8] are a well-recognized method for finding genomic areas of interest for many common complicated illnesses and phenotypes. The experiments are distinguished by analysing information acquired from large population size comprising high number (i.e., over 100K) of loci (i.e., SNPs) across the human genome. A variation at specific loci could lead to changes in biological function which may cause an illness. Such variation can be detected by analysing genotypes produced from people with and without the characteristic of interest [9].

The literature addresses a variety of approaches for assessing SNP susceptibility in GWAS where each SNP is evaluated individually [10] however, it is identified that only a small proportion of the SNPs have major impacts on the complicated disease features while, majority of the SNPs indicated low penetrance individually [11]. On the other hand, many prevalent human illnesses have been linked to intricate interactions between numerous SNPs and is referred to as multi-locus interactions [12].

In addition to conventional approaches for the GWAS analysis, Machine Learning (ML) algorithms have been utilised for identifying the SNPs that are associated to a variety of illnesses. Particularly, the ML approaches proved to be resilient when dealing with solving the non-linear problems involving high dimensional datasets similar to GWAS data used in this study. In the literature, ML techniques have been used in three major areas in the domain of genome-wide association studies [13]. Firstly, to develop classification models to distinguish between cases of disease of interest and healthy controls [14–17]. Secondly, to develop ML models to discover new genetic markers associated with a particular disease such as AD [18–20]. Thirdly, ML has been utilised to find the SNPs interactions that influence the emergence of common human diseases [21–23]. The fundamental aim for using ML in these studies is to generate prediction models that maximise the classification accuracy between cases and controls. However, the computational barrier of having hundreds of thousands of markers from GWAS data while fewer samples (i.e., data record) remains a challenge [13].

This problem has been resolved using effective feature selection methods aiming to identify the most informative variables from the available feature space. For instance, study [24] investigated the feasibility of utilising random forests (one of popular ML algorithm) for feature selection and classification on GWAS data. The findings from this work suggest that feature selection prior to data partitioning into training and testing sets, produced a model which is susceptible to overfitting. In [25], the study proposed iGnet, a deep learning model for AD classification that involves two datasets comprising MRI and genetic information. Their model combines computer vision approach to analyse the MRI scans and natural language processing to analyse the genetic data. The proposed method was evaluated over ADNI dataset indicating 83.78% classification accuracy while employing MRI data with selected SNPs from chromosome 19. Similarly, Sethi et al. [26] presents a ML model comprising convolution neural network (CNN) for automated feature extraction and support vector machines (SVM) for classification task. The main focus of the study was to develop a hybrid ML model for classification of AD using MRI data from ADNI. The hybrid model achieved better accuracy (i.e., 88%) when compared with CNN alone, with an increment of 2.9% in the model accuracy.

While the aforementioned works highlight the associations between genetic markers and AD, there are several limitations with these approaches. Firstly, conventional methods are impractical to handle the non-linearity of the complex relationships (within the GWAS dataset) for the prediction and classifications of AD. Secondly, feature selection and optimization in the existing works, is not performed in a way to be useful for the human experts (e.g., physicians, health professionals etc.) to understand the significant set of SNPs/features among the large amount of feature space. Likewise, the use of deep learning models limits the explain-ability of ML model which is not understandable by human experts.

In contrast, we propose novel wide and deep learning-based approaches to classify Cognitively Normal (CN) and AD individuals. In the first step, we conduct an association test to select the most signification SNPs influencing the disease, followed by a hybrid feature selection approach to reduce the number of features substantially. We then use a newly proposed approach of neighbouring SNPs selection, to produce a final set of SNPs which are then used for the training of wide and deep learning classification models for CN and AD subjects. Major contribution of the proposed work include:

- Developing a hybrid dimensionality reduction approach towards identification of the most distinguishing features, leading to robust classification performance.
- Propose a neighbour SNPs selection approach to test the impact of neighbour SNPs over the classification accuracy.
- Propose a wide and deep learning models for classification of individuals into CN and AD.
- Extract human understandable rules from the trained ensemble model, to serve for the machine learning model’s interpretability.

Remaining of this manuscript is organised as follows. Section 2 presents the materials and methods proposed in this study. Section 3 comprises the experimental design while Section 4 entails the results corresponding to the experimental design along with the discussions about the study outcomes.

## Materials and methods

**Fig 1:**
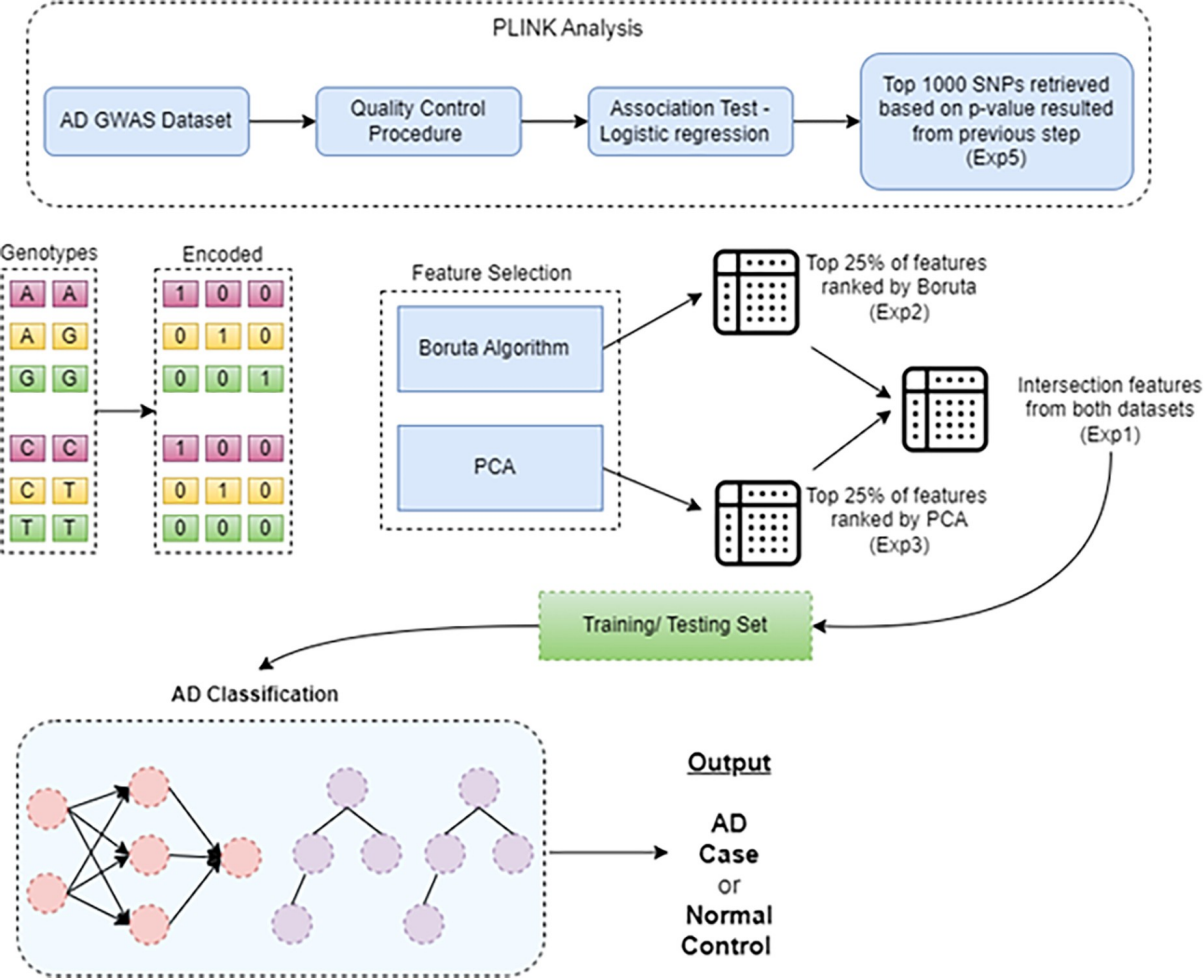
A graphical representation of proposed approach for AD and CN classification. First block represents the PLINK analysis in which quality control procedure and association test is conducted. Second the genotype data convert into one-hot representation. Third feature selected utilizing Boruta and PCA algorithms. Finally, AD classification is performed using the different feature sets.

The proposed approach for AD classification entails a composite of data processing, feature selection, and machine learning algorithms. We first perform quality control to ensure only high-quality features and samples are included. In the second step, logistic regression is used to test the association of each feature with AD. The processed dataset is then forwarded for feature selection using a hybrid approach comprising PCA and Boruta algorithms. The set of identified features are then used to train ML models for AD classification. Fig 1 shows the overall methodology of proposed AD classification where detailed implementation for each component is presented as follows.

### Dataset

Dataset used in this study is obtained from the Alzheimer’s Disease Neuroimaging Initiative (ADNI) database. The ADNI [27] was launched in 2003 as a public-private partnership with primary objective to test whether the serial magnetic resonance imaging (MRI), positron emission tomography (PET), other biological markers, and clinical and neuropsychological assessment, can be combined together to measure the progression of mild cognitive impairment and early AD.

The ADNI database comprises sets of variables including genetics, demographics and clinical data, MIR, and PET images. To fulfil the objectives of proposed study, GWAS data from ADNI1 is accessed where individuals with CN or AD were chosen. A total of 388 subjects are identified producing 174 cases and 214 controls in the proposed work.

The dataset originally is presented in plink file format with three files: ‘bim’, ‘bed’, and ‘fam’ files. In ‘fam’ file, subject characteristics are recorded. While SNPs (features) characteristics are stored in the ‘bim’ file including location, name, and allele representation. Finally, ‘bed’ files contain machine codes that are unreadable to humans and comprise 8-bit codes representing the genotype codes as well as map the information between fam and bim files. In this study, we use SNPs as features to classify the individuals into CN or AD cases. Table 1 shows the statistics of the dataset, the mean age for both cases and controls around 75 years old. the Mini-mental State Examination (MMSE) is 30-point questionnaire used measure cognitive impairment, in the utilised dataset a mode score of 23 points achieved by cases, whereas a score of around 29 achieved by controls. Table 1 also shows that most cases carry at least of copy of APOE4 gene.

**Table 1:** Characteristics statistics of Alzheimer’s disease and normal subjects.

	Age (mean)	Male/Female	Years of Education (mode)	MMSE (mode)	APOE4 (mode)	ADAS11 (mean)	ADAS13 (mean)
**Cases**	75.35	92/82	15	23	1	18.11	26.99
**Controls**	75.66	115/99	16	29	0	5.83	8.98

### Quality control

To filter out unnecessary information from both genetic markers and samples, several techniques have been studied and used in genetic data quality control, with an emphasis on SNP data. The methods described in this section are best practises for removing individuals and SNP traits that might induce bias, impede or mask signals, or produce false positive results [28]. The dataset prepared in the proposed study is reduced to a representative set of SNP characteristics and subject cohort that are more likely to exhibit underlying genetic signals in conjunction with the phenotype; by eliminating subjects and SNPs that do not meet the requirements imposed by these procedures. Originally, there are 6,20,901 number of SNPs that are reduced to 4,87,037 SNPs using operations described in Table 2.

**Table 2:** Quality control procedure applied for both samples and genetic markers.

Filtering approach	Description	Threshold Used
SNPs missingness	Missing SNPs in a large percentage of the Individuals are excluded.	0.02 genotyping rate
Individuals’ missingness	Individuals with a high rate of genotype missingness are excluded.	0.2 genotyping rate
Sex discrepancy	Check sex of individuals depending on their X chromosome homozygosity	An estimate of the X chromosome homozygosity > 0.8 for males and <0.2 for females.
Autosomes Chromosomes	Only selecting SNPs of 1 to 22 Chromosomes	-
Minor allele frequency	SNPs above a minor allele frequency threshold are included.	0.05 due to sample size.
Hardy–Weinberg equilibrium (HWE)	SNPs that deviate from HWE are excluded.	SNPs are first filtered out within the controls for HWE p-values of 1e-6, then in cases for HWE with p-value of 1e-10.
Relatedness	Generates a list of persons with relatedness degree greater than a specified threshold.	employ 0.2 pi-hat threshold. After including only founders, three pairs were discovered. We eliminate the person with the lowest call rate.
Population stratification	Individuals from different populations present in the study.	Only non-Hispanic European participants chosen.

### Association test

**Fig 2:**
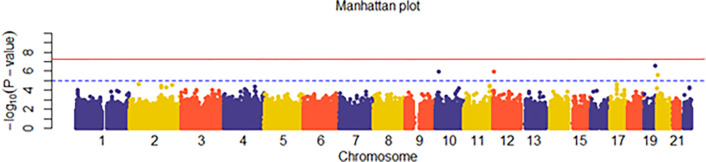
Manhattan plot of GWAS between Alzheimer’s disease and normal controls.

**Fig 3:**
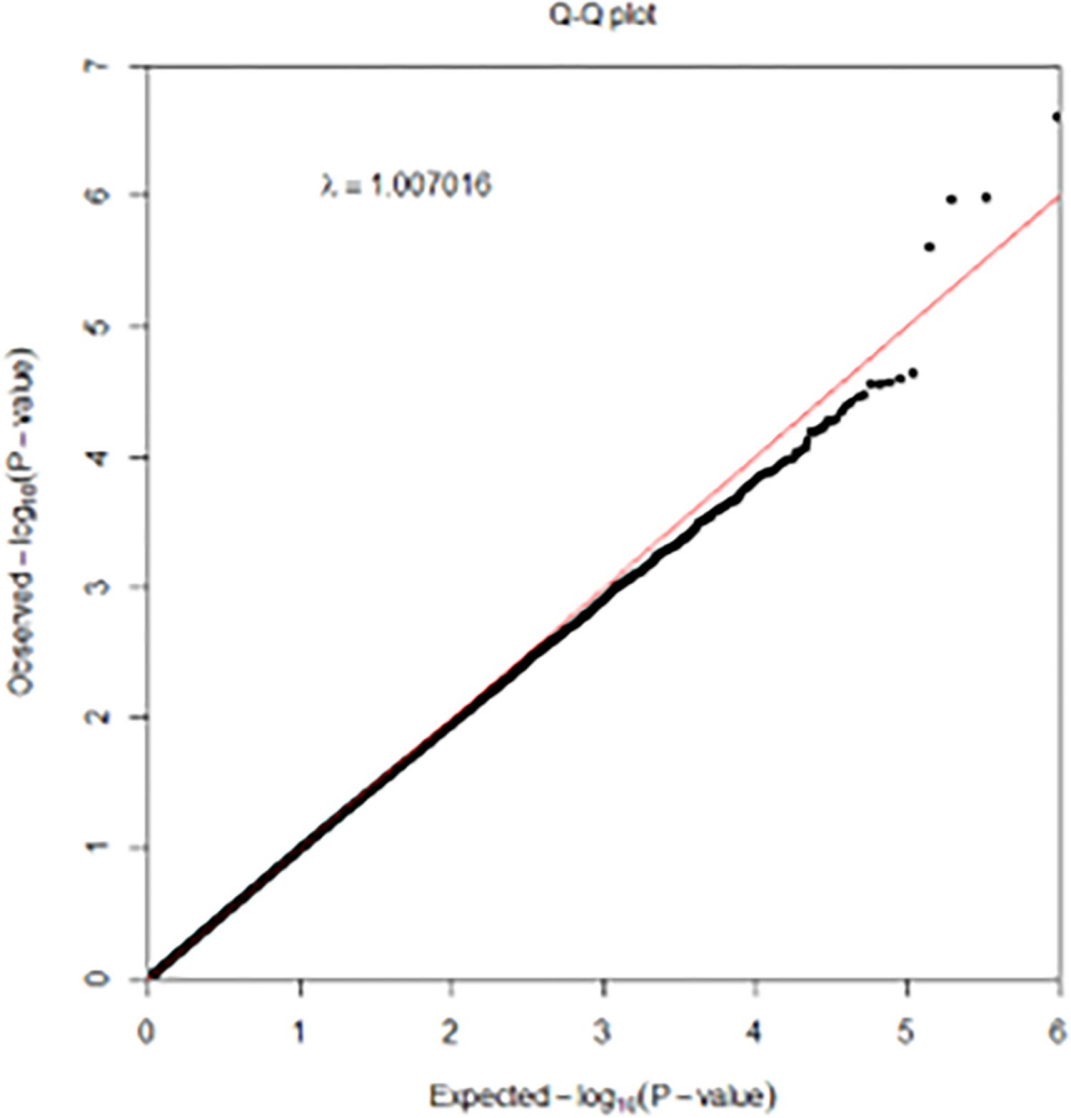
QQ plot of GWAS between Alzheimer’s disease and normal controls. Lambda is close to 1 which means the points falls within the expected range.

In case-control studies, the frequency of alleles or genotypes at SNP differs between cases and controls in a particular population. We use the associations tests to identify the statistically significant variations in the frequency of alleles across research participants. These alleles are used to test for phenotypic relationships. In other words, association analysis is a set of single-locus statistical tests that investigate each SNP and its potential connection with a certain trait [29]. In this context, logistic regression is one of the common methods which has been used in similar works [30, 31] for studying each SNP individually and capturing the linear associations between SNPs and phenotypes. Analysis GWAS data is challenging due to the high dimension of features which, comprise hundreds of thousands of SNPs. To overcome this, we utilise an association test for each SNP, producing significance of association (i.e., p-value) with AD. GWAS [32] utilises an approximation where significant relationships have a p-value less than 5*10^−8^, even if a greater number of genetic variants are examined. Such statistically meaningful results can only be obtained by studying large samples (about 1000 individuals or more). Therefore, we have selected top 1000 SNPs according to the lowest p-value of logistic regression. On a genomic scale, Manhattan plots depict the p-values of whole GWAS (see Fig 2). The P values are given in genomic order by chromosome and chromosomal location (x-axis) where y-axis value shows the log10 of the p-value. In addition to Manhattan plot, the Quantile-Quantile (QQ) plot is a graphical depiction of the observed p-values’ divergence from the null hypothesis: observed p-values for each SNP are ordered from biggest to smallest and shown against predicted values. If the observed values match the predicted values, all points land on or near the centre line connecting the x- and y-axes (null hypothesis: red line in Fig 3). Therefore the data is normally distributed.

Following the association test, the genotype data is transformed to binary representation using one-hot coding [33] and used as input to feature selection algorithms and ML models. Genotypes of each SNP is converted into a three-dimensional vector replacing ‘1’ for the genotype and 0 for the other two as shown in Fig 1. As an example, vector [CC, CT, TT] is transformed into [1,0,0], [0,1,0], and [0,0,1], respectively.

### Feature selection

Large datasets such as GWAS, have been gaining popularity in human disease research however, multi-attribute analysis and complex inter-relationships within multi-dimensional datasets, are difficult to be performed using conventional data analysis approaches. Such challenges limit the usefulness of these datasets. To overcome this challenge, feature selection has been reported useful particularly for the dimensionality reduction in such datasets. The reduced set of features preserving the maximum proportion of information from the original feature space, is useful for the simplicity of machine learning model. As a result, it is increasingly used in many real-world applications, such as gene analysis [34], to obtain relevant features by eliminating the useless and redundant information. This furthermore reduces the computational and storage costs and improving the model’s learning performance [35].

For the feature selection and dimensionality reduction in proposed work, we firstly conducted an association test using logistic regression (as described in Section Association Test) to calculate the association of each SNPs with the AD. The top 1000 SNPs based on corresponding significance values (i.e., p-value) are retrieved for further analysis. The selected 1000 SNPs are then feed to a composite of feature selection approaches that include Principal Component Analysis (PCA) [36] and Boruta algorithm [37], which has been used in various similar domains [38, 39]. Details of each feature selection method is presented in the following sections.

#### a) Principal component analysis

**Fig 4:**
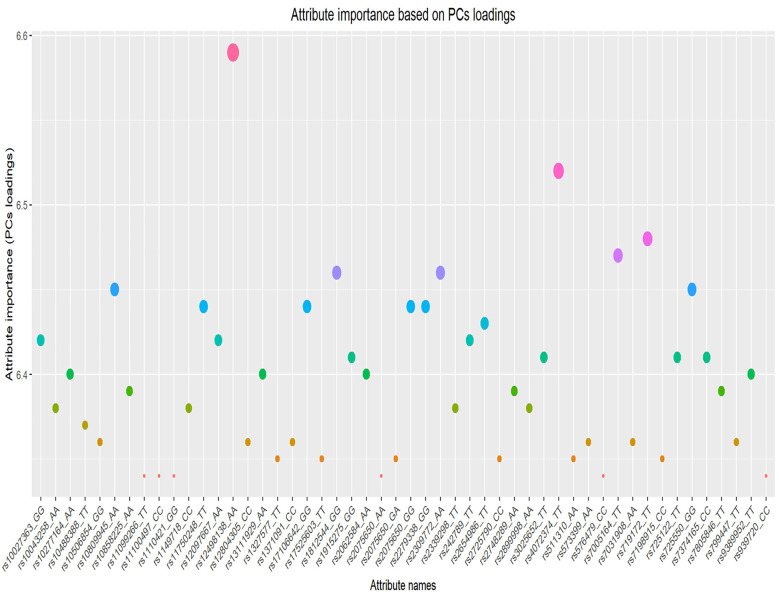
Top-ranked 50 features (out of 1000 SNPs) selected as important, by the PCA algorithm.

Principal component analysis is one the powerful statistical method which have been successfully employed in various research studies mainly, for the dimensionality reduction and feature selection [37]. The main idea behind PCA is straightforward: reduce the number of variables in a data set while retaining information as much as possible. This entails identifying new variables that are linear functions of attributes in the original dataset, maximise variance sequentially, and are orthogonal to each other. The transformed variables are known as principal components (PCs) [40]. In our case, the component loadings represent correlation coefficients between SNPs where maximized sum of variances of the squared loadings is retrieved through the components’ rotations. Importance measure for the corresponding features in original space (i.e., dataset) can be calculated using the absolute sum of component rotations [39]. The top-ranked 50 features (out of 1000 SNPs) selected by the PCA algorithms (as most important) are shown in Fig 4, including rs12498138 located on gene GOLGB1, rs4072374 located in gene RNASEH1, rs2309772 in TENM3, rs7005164, and gene LOC105375901.

#### b) Boruta algorithm

The Boruta algorithm is a wrapper method that is based on the Random Forest (RF) classification algorithm. The Boruta algorithm use selection criteria for important factors by eliminating variables that are statistically identified as less relevant than random probes iteratively. Detailed implementation of the Boruta algorithm can be found in study [37]. In the proposed study, SNPs with substantially high scores identified by the Boruta algorithm includes: rs17365991 gene TEF, rs8141950 gene PARVB, rs2654986 gene LUNAR1, and rs2036109 gene ADRA1A. A complete list of important features selected by the algorithm is presented in S1 Table in S1 File.

#### c) Hybrid feature selection

While both PCA and Boruta algorithms are commonly used for the feature selection, the fundamental of mathematical formulations of both algorithms is different. Combining the outcomes form both algorithms might be useful to filter-out maximum number of features while simultaneously, retaining maximum information from the original dataset. For this purpose, we perform a hybrid feature selection as a composite of Boruta and PCA outcomes. In the first step, outcomes from both feature selection algorithms are sorted with respect to feature ranks (i.e., feature importance). We then selected the intersection of 1^st^ quartiles of features (i.e., top 25%) that are identified from both Boruta and PCA algorithms, producing 121 of most significant features. A complete list of the commonly selected features is presented in Table 3. It can be noticed that some of the top-ranked SNPs located in genes are strongly related to AD such as rs6116375 on gene PRNP, rs2075650 on gene TOMM40.

**Table 3:** List of final feature-set identified as significant using the intersection of selected features from both PCA and Boruta algorithm.

rs6116375_CC	rs10176603_TT	rs7747741_GG	rs4290760_CC	rs16864809_TT
rs2654986_TC	rs10031325_CC	rs701880_CC	rs11680332_GG	rs7679260_CC
rs11768384_GG	rs16889565_GA	rs9296691_TC	rs628482_GG	rs9389952_TT
rs2075650_AA	rs2877347_CC	rs4953672_CC	rs518385_TT	rs10804812_CC
rs7342676_CC	rs6114605_GA	rs10068900_GG	rs2577322_CC	rs618236_CC
rs4964453_TT	rs7618348_CC	rs2834714_TT	rs11869174_CT	rs1945624_AA
rs10790928_TT	rs9595108_CC	rs6838005_CC	rs11733633_AA	rs2577322_TT
rs2208322_AA	rs17068548_GG	rs10514486_CC	rs911892_TT	rs7807731_TT
rs7519796_AA	rs13211072_TT	rs7149949_TT	rs3812568_AA	rs2136613_TT
rs10222715_TT	rs6132022_TT	rs2725790_CT	rs799447_GG	rs344783_TT
rs10793982_TT	rs793291_AA	rs11655031_TT	rs17745021_CT	rs1495813_CC
rs775879_GG	rs3771389_CT	rs2833427_CC	rs13245564_GG	rs9410486_GG
rs4837137_AA	rs6695731_CC	rs8007000_TT	rs2305252_AA	rs7096762_AA
rs1789250_AA	rs10044783_CC	rs17430865_CT	rs4472075_AA	rs2309777_GG
rs4868468_AA	rs17345545_CC	rs3815360_CC	rs4793902_TT	rs9515168_GT
rs11752811_TT	rs871049_CC	rs17430865_TT	rs168825_GG	rs6569364_AA
rs2075650_GG	rs4953672_AA	rs11922179_AA	rs6838005_TC	rs12988856_TT
rs2697303_AA	rs2075650_GA	rs1186685_TT	rs775879_AA	rs1891265_GG
rs362584_AA	rs1479884_GG	rs7320494_AA	rs6903956_AA	
rs8000805_GG	rs11253696_AA	rs7206002_GG	rs12480224_AA	
rs10879839_TT	rs13135230_GG	rs367369_TT	rs2339298_TT	
rs2286343_AA	rs10888578_TT	rs1328179_TT	rs7413155_AC	
rs939720_CC	rs7999171_GG	rs4689705_TT	rs9595108_AC	
rs7165661_TT	rs12312628_CC	rs705904_CC	rs6929400_CC	
rs2867922_TT	rs10101666_TT	rs9381936_CC	rs268909_TT	

The aforementioned features (PCA, Boruta, and composite of both) are then used to train and validate the multiple ML models for the task of AD classification over unseen instances.

### Proposed Alzheimer’s disease classification

Once the most promising features are identified from the original dataset, we then employ multiple well-established classification methods, to classify AD that include RF, artificial neural networks (ANN), and deep ANNs. For the detailed experimental analysis, we use variations of inputs (i.e., feature combinations) to selected models for efficient classification of AD along with identification of significant set of features. A detailed description of each classifier with respect to proposed work, is presented as follows.

#### a) Random forest for proposed AD classification

Ensemble learning is an effective technique for combining multiple learning algorithms to improve overall prediction accuracy. These ensemble techniques have the advantage of alleviating the problem of small sample size by averaging and incorporating over multiple classification models, to reduce the possibility of overfitting the training data. As a result, the training dataset can be used more efficiently, which is important in many biological applications with small sample sizes. Some ensemble methods, such as RF, are particularly useful for high-dimensional datasets because generating multiple prediction models, each with a different feature subset, can improve classification accuracy [41].

**Fig 5:**
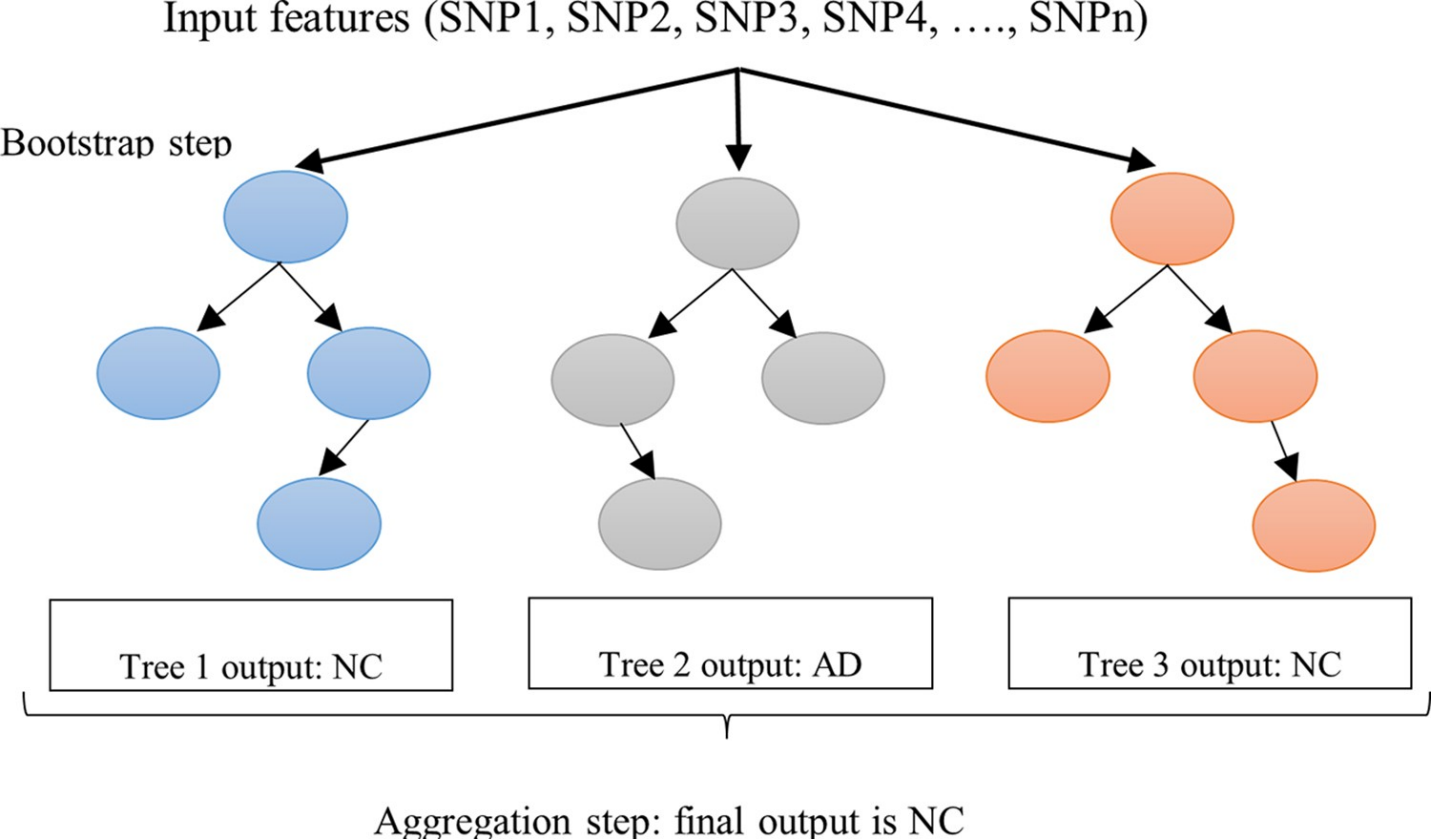
Random Forest sub-trees for proposed AD classification using GWAS data. The input to the RF is the bootstrapped SNPs features. In the first step (bootstrap step) refers to the process of training each tree in RF on a subset of the training samples. While in the second step (aggregation step) the class with the majority votes from the trees is chosen as the final output (in above example 2/3 votes are in favour of Normal control).

Recently, RF has been successfully employed in diverse application areas for both classification [42] as well as regression problems [43]. Generally, RF is made up of several decision trees with the principle of bagging, which combines the operations of bootstrapping and aggregation. Bootstrapping refers to the process of training each decision tree on a subset of the training samples, utilizing a subset of the original features, ensuring that each tree is distinct, which significantly helps in overcoming the problem of the classifier’s variance. Within the aggregation step, the output of each tree is considered, and the class with the majority votes from the trees is chosen as the final output. Further details on RF can be found in related work [44]. Fig 5 depicts a sample of single decision tree (with bootstrapped data sample) from the proposed RF-based AD classification model.

#### b) Deep wide artificial neural networks for proposed AD classification

Similar to RF, feed-forward neural networks have been successful in variety of applications within diverse disciplines [45–47]. It consists of a network of linked neurons with linear or nonlinear transfer functions that may be used to analysis nonlinear data such as genetics in this study. With only two layers of neurons, a feed-forward neural network may estimate sensible functions to any desired degree of precision.

Based on the theoretical concepts in [48], we employ a neural networks with gradient descent optimization utilising the backpropagation learning approach for binary classification problems. The neural network is built using input, hidden and output layers that each include a predetermined number of units (neurons). Various neural networks architectures are employed in the current work: a Wide Neural Network (WNN) which consist of one hidden layer with a large number of neurons and Deep Neural network (DNN) consisting of multiple hidden layers with smaller number of neurons in each layer.

**Fig 6:**
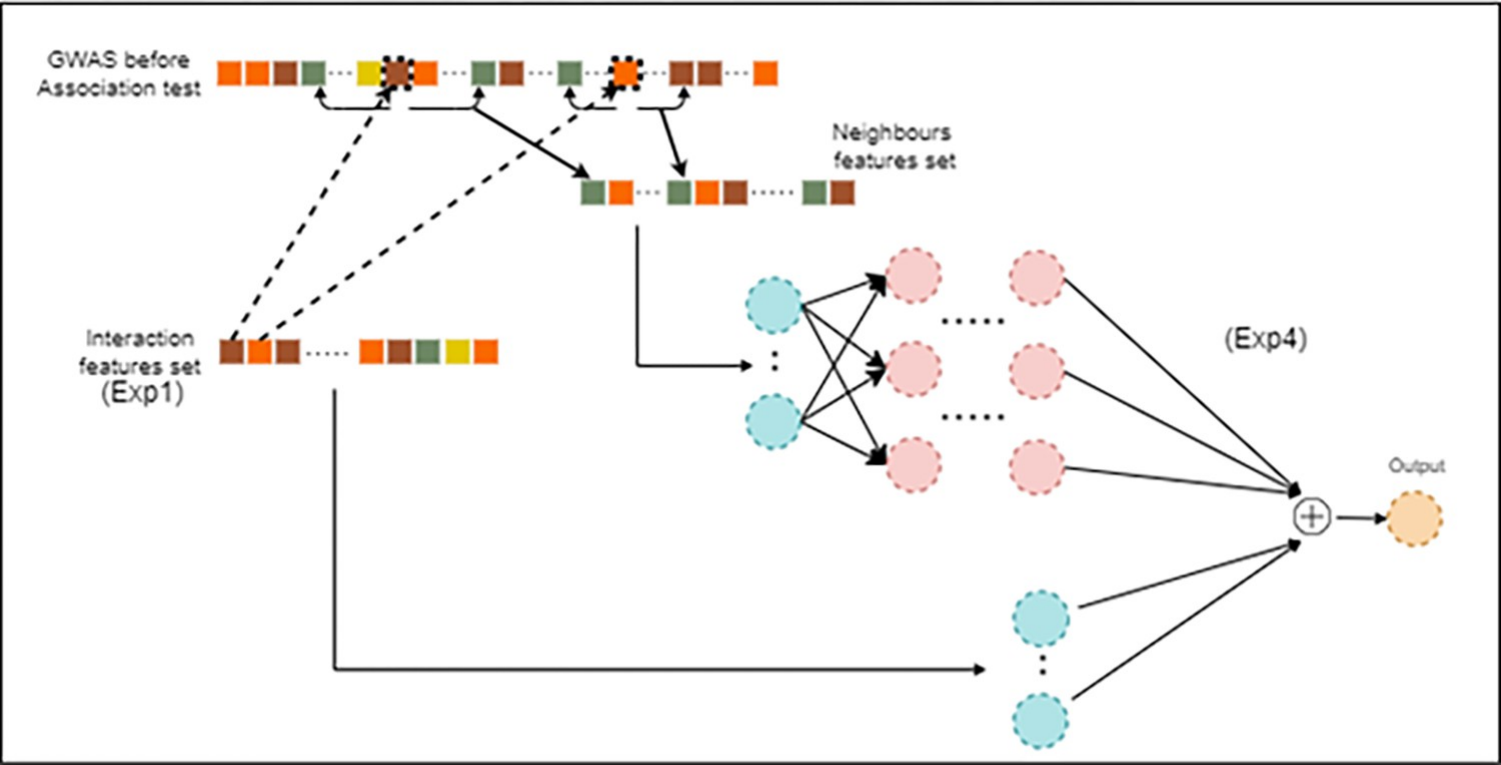
Proposed Wide and deep NN for AD classification using GWAS data.

Furthering the artificial neural network concept, a wide and deep neural network (illustrated in Fig 6) is a combination of a deep neural network and a linear model based on a small set of features. Deep learning tends to generalise data patterns, whereas linear models help to learn the patterns. This type of architecture has been reported useful in similar works such as cell type classification [49] and recommender systems [50]. The deep component of the network can handle the high-dimensional data, whereas the wide component emphasises the biological significance of SNPs to AD, by integrating them into the network’s final hidden layer. For the proposed AD classification (as illustrated in Fig 6), the final set of identified features (Table 2) are fed to the wide component. For each SNP identified in Table 2, we retrieved neighbouring SNPs which are then served as an input to the deep component of the network.

### Experiment design

Multiple experiments are conducted using the identified features via proposed hybrid feature selection (see Section Feature Selection) from the ADNI GWAS dataset, to train the aforementioned AD classifiers (RF, WNN, and DNN). For the training and testing, we partition the dataset into 70% and 30% respectively. We further employ the cross-validation (5-CV) for a fair and reliable evaluation of the proposed AD classifiers’ performances. For all ML classifiers, the optimal set of hyperparameters are determined using the trial-and-error method and are detailed in S2 Table in S1 File. Quality control steps and association test are conducted using PLINK software [51] while ML algorithms are implemented using Scikit-learn python library [52]. PyPlink library is used to read the genotype data in python [53]. Finally, the neural networks implementation is performed with Keras and TensorFlow as backend [54]. With these configurations and feature sets, following set of experiments are performed in the proposed study:

Experiment 1 (EXP1): Using intersection of 1^st^ quartile (i.e., top 25%) of features that are ranked from both Boruta and PCA algorithms. The combined feature set (called Intersection feature set) is used to train ML algorithms (RF, WNN and DNN) in order to find the best performing GWAS AD classifier.
Experiment 2 (EXP2): Using top 25% of features ranked by Boruta extracted as a feature set (called Boruta feature set) for the AD classification using RF, WNN and DNN algorithms.
Experiment 3 (EXP3): Using top 25% of features ranked by PCA extracted as a feature set (called PCA feature set) for the AD classification using RF, WNN and DNN algorithms.
Experiment 4 (EXP4): In order to evaluate the effect of neighbouring SNPs, for each SNP in the interaction features set (EXP1), we retrieved the SNP and neighbouring SNPs (6 from each side) and constructed a new feature space, called neighbouring features set. Using features from EXP1 as input to the wide component and neighbouring features set as input to the deep component to train and test the proposed wide and deep model (As shown in Fig 6).
Experiment 5 (EXP5): Using top 25% of features of the logistic regression extracted as a feature set (called original feature set) for the AD classification using RF, WNN and DNN algorithms.

#### Performance evaluation

Performances of the AD classification models is evaluated using several standard evaluation metrics. Model accuracy (Eq 1) describes how well the model performs across all classes. Using precision (Eq 3), we determine how many predictions of positive classes are actually positive. Recall (Eq 2), as opposed to precision, indicates how many positive predictions were missed. The F-score (Eq 4) is calculated by averaging precision and recall determining the classifier’s accuracy. Furthermore, Receiver Operating Characteristic curves (ROC) is used as a performance measurement of classification efficiency at different thresholds. With a higher Area Under the Curve (AUC) value, the model is more effective at making a distinction between cases (i.e., patients) and controls (i.e., healthy subjects). Finally, Precision-recall curves (PR) show the trade-off between precision and recall w.r.t. varying thresholds.


$$Accuracy=\frac{TP+TN}{TP+TN+FP+FN}$$ (1)

$$Recall=\frac{TP}{TP+FN}$$ (2)

$$Precision=\frac{TP}{TP+FP}$$ (3)

$$F-score=\frac{2*Precision*Recall}{Precision+Recall}$$ (4)

## Results and discussion

Following the aforementioned experimental configurations, detailed statistical results and performance measures are retrieved. Particularly, this study is first of its kind to identify and extract the most promising (as well as substantially reduced in quantity) set of features which significantly contribute to classification of AD. We identify a number of genes as significantly related to the AD that are aligned with related literature including rs6116375 on gene PRNP [55], rs2075650 on gene TOMM40 [56], rs10793982 on gene LAMC3 [57], rs2208322 on gene NEURL1 and rs7519796 on gene KAZN [58], demonstrating the efficacy of our features selection approach. Furthermore, we identify some of the potential novel SNPs such as rs2654986 on gene LUNAR1, and rs2208322 on gene NEURL1 that are significantly associated with AD. A complete list of the significant SNPs identified in proposed study is available in Table 3.

To evaluate the effectiveness of our feature selection process, a RF classifier and ANN with varying parameter configurations are employed to classify the AD patients. The performance of the classifiers is presented in Table 4 when evaluated over the unseen subjects using features set described in EXP1. It can be noticed that regardless of selected ML model, high performance measures are achieved. WNN indicates an accuracy and f1-score of 94% and 93% respectively followed by a DNN which showed a slightly decline in performance (i.e., 93%). While RF indicate more deteriorations in performance with 89% accuracy and 88% F1 score, which is in line with the existing similar work [59], where higher accuracy is reported using ANN as compared to RF (for preterm birth classification). Oriol et al. [15] employed RF in classification of AD and CN using GWAS data, where they reported accuracy of 67% (significantly lower than proposed approach). Similarly, RF was not the best classifier to discriminate between AD cases and controls as reported in a similar work [60]. It is also important to note the performance balance from WNN and DNN (in Table 4) as compared to RF, which indicates more biasedness towards the precision (96%) as compared to recall (81%). The accuracy and loss curves of the models are available in S1 and S2 Figs in S1 File.

**Table 4:** Comparison of ML algorithms for classification of AD and healthy individuals using intersection features selected by Boruta and PCA from the top 25% (Exp 1).

Model	Accuracy	Precision	Recall	F1
**RF**	89%	96%	81%	88%
**Wide NN**	94%	91%	98%	93%
**Deep NN**	93%	89%	96%	92%

Table 5 summarises outcomes for EXP2 where all classifiers indicated similar performance when trained and tested over the top-ranked (i.e., 1^st^ quartile) features selected by Boruta algorithm. It can be noticed that the overall accuracy of each model is increased specifically, the WNN and DNN which indicate 99% accuracies for unseen instances. This clearly indicate the effectiveness of selected features as well as the model’s configurations.

**Table 5:** Comparison of ML algorithms for classification of AD and healthy individuals using top 25% features selected by Boruta algorithm (Exp 2).

Model	Accuracy	Precision	Recall	F1
**RF**	92%	99%	84%	91%
**Wide NN**	99%	99%	99%	99%
**Deep NN**	99%	99%	99%	99%

Table 6 presents the outcomes for EXP3 where the features identified from PCA algorithm are used to train the ML models. It can be noticed that WNN and DNN models outperformed the RF producing overall 96% and 94% accuracies as compared to 84% from RF. Likewise, the performance clearly indicates the balance between recall and precision which is not the case for RF. Overall, in comparison, the RF demonstrated a notable reduction in performance.

**Table 6:** Comparison of ML algorithms for classification of AD and healthy individuals using top 25% features selected by PCA algorithm (Exp 3).

Model	Accuracy	Precision	Recall	F1
**RF**	84%	99%	68%	81%
**Wide NN**	96%	99%	92%	96%
**Deep NN**	94%	96%	91%	93%

To assess the impact of the neighbouring SNPs (of the identified most important SNPs) towards the classification of AD, we evaluated the performance of WDNN classifier in EXP4 (Table 8). Despite the performance of WDNN is substantially reduced (around 80%) as compared to EXP1-EXP3, it is still inline or outperforms most of the existing related works as shown in Table 8, particularly in the domain of GWAS. For the final experiment, we tested the models’ performances over the original dataset (EXP5 as illustrated on Fig 1) before feature selection (Table 7). It can be noticed that the classification performance from each model is nearly as accurate as in EXP2 (Table 4). Likewise, the RF indicates a biased performances in terms of precision and recall.

**Table 7:** Comparison of ML algorithms for classification of AD and healthy individuals using original features set (Exp 5).

Model	Accuracy	Precision	Recall	F1
**RF**	91%	99%	81%	89%
**Wide NN**	99%	99%	98%	99%
**Deep NN**	99%	99%	98%	98%

### ROC and PR curve analysis of the AD classifiers

**Fig 7:**
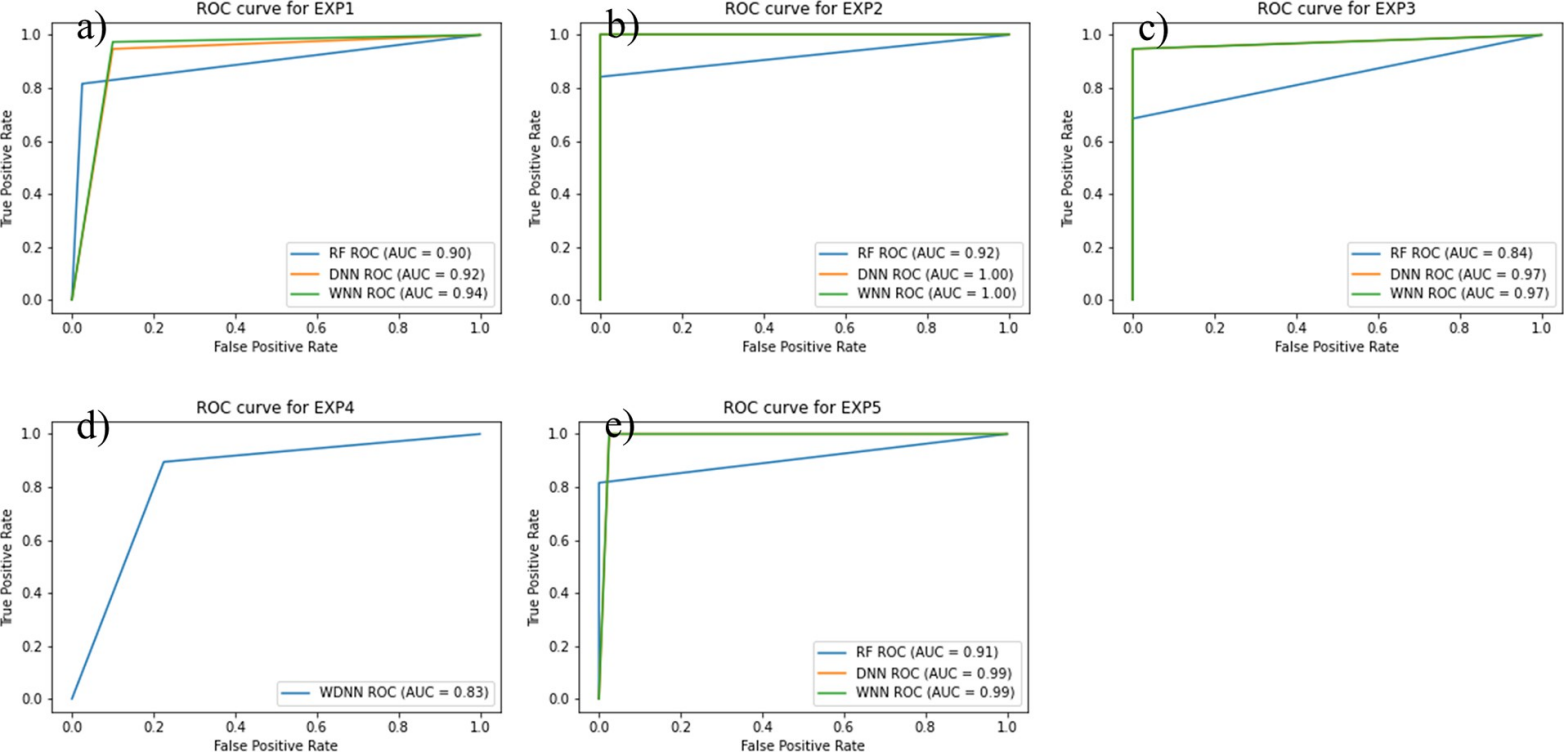
(a) ROC-AUC curve for EXP1, (b) ROC-AUC curve for EXP2, (c) ROC-AUC curve for EXP3, d) ROC-AUC curve for EXP4, (e) ROC-AUC curve for EXP5.

**Fig 8:**
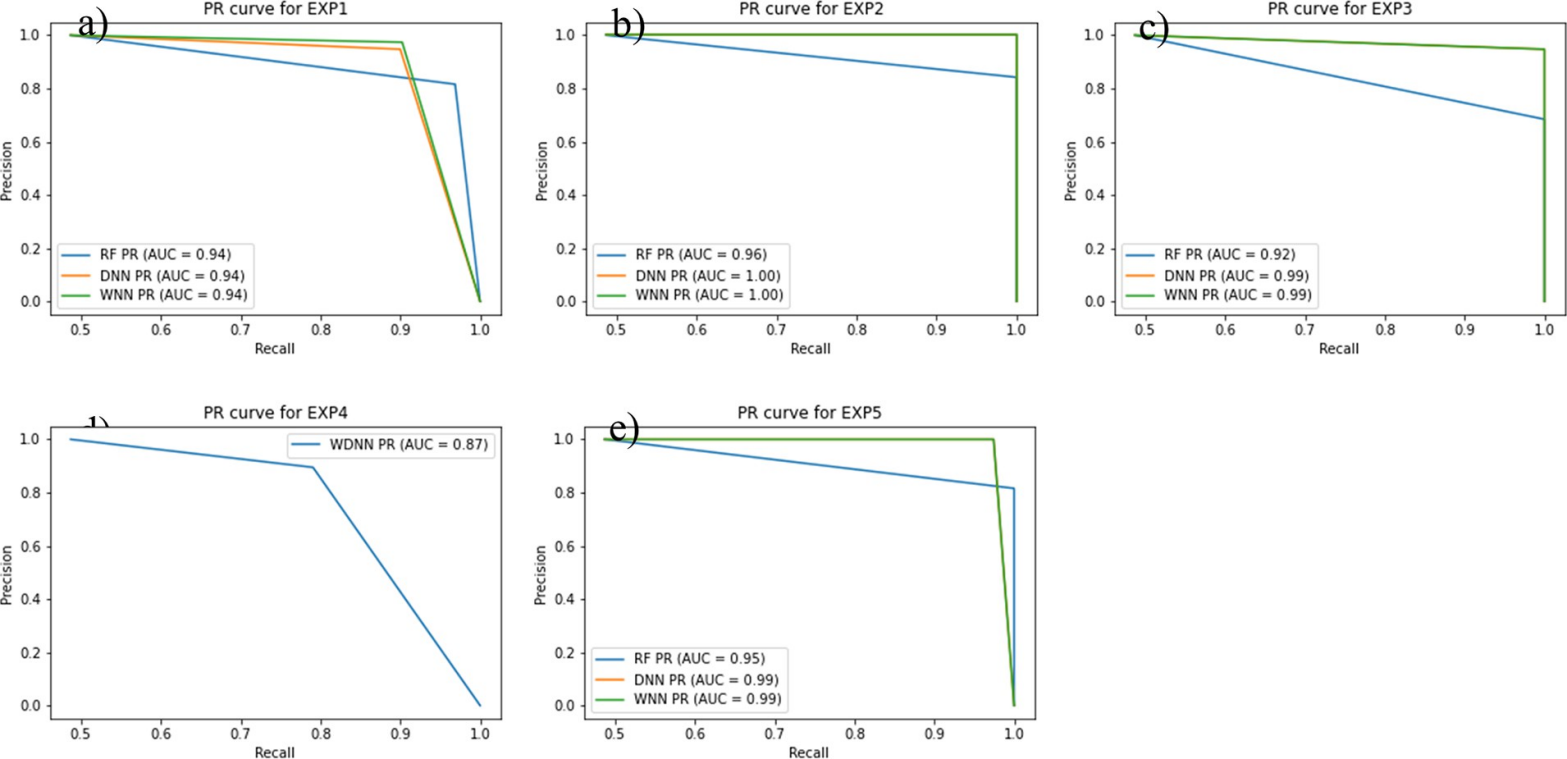
(a) PR-AUC curve for EXP1, (b) PR-AUC curve for EXP2, (c) PR-AUC curve for EXP3, d) PR-AUC curve for EXP4, (e) PR-AUC curve for EXP5.

Figs 7 and 8 show the ROC and PR curves of the ML classifiers’ performances in classifying AD cases and normal controls. From Fig 7, we can clearly see that WNN and DNN performed similar in most of the experiments. WNN is the most sufficient classier for AD classification reaching 100% in terms of both ROC and PR (Fig 7B) when tested on feature selected by Boruta algorithm (EXP2). In case of classifiers’ training evaluation over the intersection feature set (i.e., EXP1), a performance of 90% or over is achieved in terms of AUC for both ROC and PR curves (Figs 7A and 8A). The WDNN model (EXP4) shows an 83% (Fig 7D) and 87% (Fig 8D) AUC for ROC and PR, respectively. These results indicate the efficiency of Boruta algorithms for feature selection that are useful to detect and classify the AD in individuals.

### Comparative analysis

Finally, Table 8 compares the performance of proposed method with existing similar approaches, towards the classification of AD based on genome-wide data (SNPs). It is evident that our approach outperforms the Decision tress [60], CNN [61], ensemble models [15], and LASSO [62]. The proposed approach shows stable performance throughout the evaluation metrics including ROC. Whereas, the decision tress utilised in reference [60] showed an increase AUC of 11% comparing to the model’s accuracy. Likewise, our work shows the superiority of Boruta algorithm in selecting the optimal number of features and eliminating the redundant SNPs, which reflects the high performance in the classification task. The results indicate that Boruta algorithm is better than other feature selection techniques such as statical techniques applied in [62]. Moreover, the proposed model uses only 121 features as input to the WNN as compared to state-of-the-art methods such as [62] which uses over 500 features, and CNN-based approach utilising 400 features [61]. This leads to a less noisy, lighter, and more efficient model as proposed in this study. The identification of fewer contributing features to AD may be useful to set a baseline for further analysis and direction in future research.

**Table 8:** Comparison of related work in the literature.

**AUC**	91%	81%	72%	84%	94%	100%	83%
**Prec**	80%				91%	99%	79%
**Recall**	80%		70%	82%	99%	99%	89%
**F score**	80&				95%	99%	83%
**Acc**	80%	75%	~70	84%	95%	99%	83%
**Feature No.**	145	4000	2500	501	121	747	121 for wide component and 4697 for deep component
**Feature selection**	Previously reported SNPs related to AD from DiaGeNet database.	Divided the genome into nonoverlapping fragments, then used CNN to select segments. CNN was run on the selected fragments using a Sliding Window Association Test to identify important SNPs.	To find significant SNPs, used the statistical summary results from IGAP [23]. The top 2,500 SNPs were then chosen as the final feature set.	Using X2 with kinship correction	See section 3.	See section 3.	See section 3.
**Dataset**	ADNI3	ADNI	ADNI	NIA-LOAD	ADNI	ADNI	ADNI
**ML Model**	Gradient boosted decision trees	1D CNN	Ensemble of ML models	LASSO	WNN (EXP1)	WNN (EXP2)	WDNN (EXP4)
**Study**	**[60]**	**[61]**	**[15]**	**[62]**	Proposed Model 1	Proposed Model 2	Proposed Model 3

## Discussion

First of all, to the best of authors’ knowledge, the study is first of its kind to examine GWAS data using a wide and deep neural network approaches. Secondly, using a relatively small number of identified feature set (only 121 features) using proposed feature selection approach, the classifying models achieved outstanding performance (Table 4), which reveals the robustness of our feature selection methodology. Furthermore, experimental outcomes show that using appropriate classifier can improve the accuracy better than increasing the number of features (See Table 5). In addition to performance efficiency, experiments 1,2 and 3 show the strength of neural networks in the existence of complex relations within the dataset. The results demonstrate the effectiveness of our approach (e.g., via the cross validations) which can be easily applied to other chronical disease where larger GWAS datasets are available.

Similar to other related studies, when interpreting the findings, some limitations are also noticed in the proposed work. Firstly, the sample size is relatively small however, this is consist with other related work that uses the same dataset [15, 17, 62–64] and other work which use GWAS data with a similar or lower sample size [65, 66]. Secondly, number of features (SNPs) highly exceeded the number of samples within the original dataset however, we addressed this issue by substantially reducing the number of features using advanced statistical approaches and highlighted the significant SNPs.

We also conducted experiments to compare the performance of WNN (one hidden layer with a large number of neurons) and DNN (multiple hidden layers with smaller number of neurons in each layer) to explore the implication that architecture selection has in the model performance. The ANNs have variety of parameters to choose from, including the number of hidden layers and neurons per layer. These parameters distinguish the network’s architecture and influence how the model performs. We noticed that in almost all of our experiments, WNN outperforms the DNN that may be because of the size and nature of the dataset. More interactions between input variables can be approximated by WNN where DNN are commonly used in computer vision and natural language processing problems.

Furthermore, it can be noticed that the WNN and DNN showed better performance than RF in GWAS domain (Tables 4–6). However, there is a trade-off between model accuracy and model interpretability. The RF can lead to an interpretable model and extract useful explanation on how the model reached a decision (case or control) which to go beyond simply using a model to get the best possible predictions. The RF model can produce insights which a human expert (e.g., physicians) can use to understand how the model help in AD diagnosis through genetic data. For this purpose, a list of human understandable rules is extracted from the best performing tree of our RF model as shown in supplementary materials (S3 Table in S1 File).

From the extracted rules, we can infer that if a person has the genotype of CC for SNP rs705904 and GG for SNP rs799447 or AA for SNP rs11922179, they are less likely to be diagnosed with AD. Furthermore, genotype of AA for SNP rs2075650 is highly associated with controls. On the other hand, a person with genotype AA for SNP rs1789250 or genotype other than AA for SNP rs2075650 is most likely to be a case of AD.

## Conclusion

In the current study, we requested access to human genome wide data from AD neuroimaging initiative, in order to build a reliable machine learning classifier to classify patient with AD and normal controls. Both of Boruta and PCA algorithms utilized as feature selectors to reduce the number of features and identify the most promising set of SNPs. We then conduct detailed experiments, by training the machine learning models on different features subsets. Wide and deep learning approaches proposed for classifying AD and non-AD subjects. All models achieved high performance; wide neural network found to be the best classifier with a stable performance of 99% accuracy. The outcomes clearly demonstrate the effectiveness of proposed hybrid feature selection. Based on our findings, there are several future works we recommend within the study context. Larger dataset can be used to examine the generalization of these models. Further analysis is required to investigate the associations of the identified SNPs with AD. Although of the models used to classify AD patients it can be extended to other chronic disease.

## Supporting information

S1 File (PDF)
