# Supplementary material for: Wide and deep learning based approaches for classification of Alzheimer’s disease using genome-wide association studies
Source: PLoS One. 2023 May 1;18(5):e0283712. doi: 10.1371/journal.pone.0283712 (PMC10150974; doi:10.1371/journal.pone.0283712)
Supplement: S1 File — (PDF) [file pone.0283712.s001.pdf]

Wide and Deep Learning Based Approaches for Classification of Alzheimer's Disease Using Genome-Wide Association Studies

Abbas Alatrany, Wasiq Khan, Abir Hussain and Dhiya Al-Jumeily

Supplementary Materials

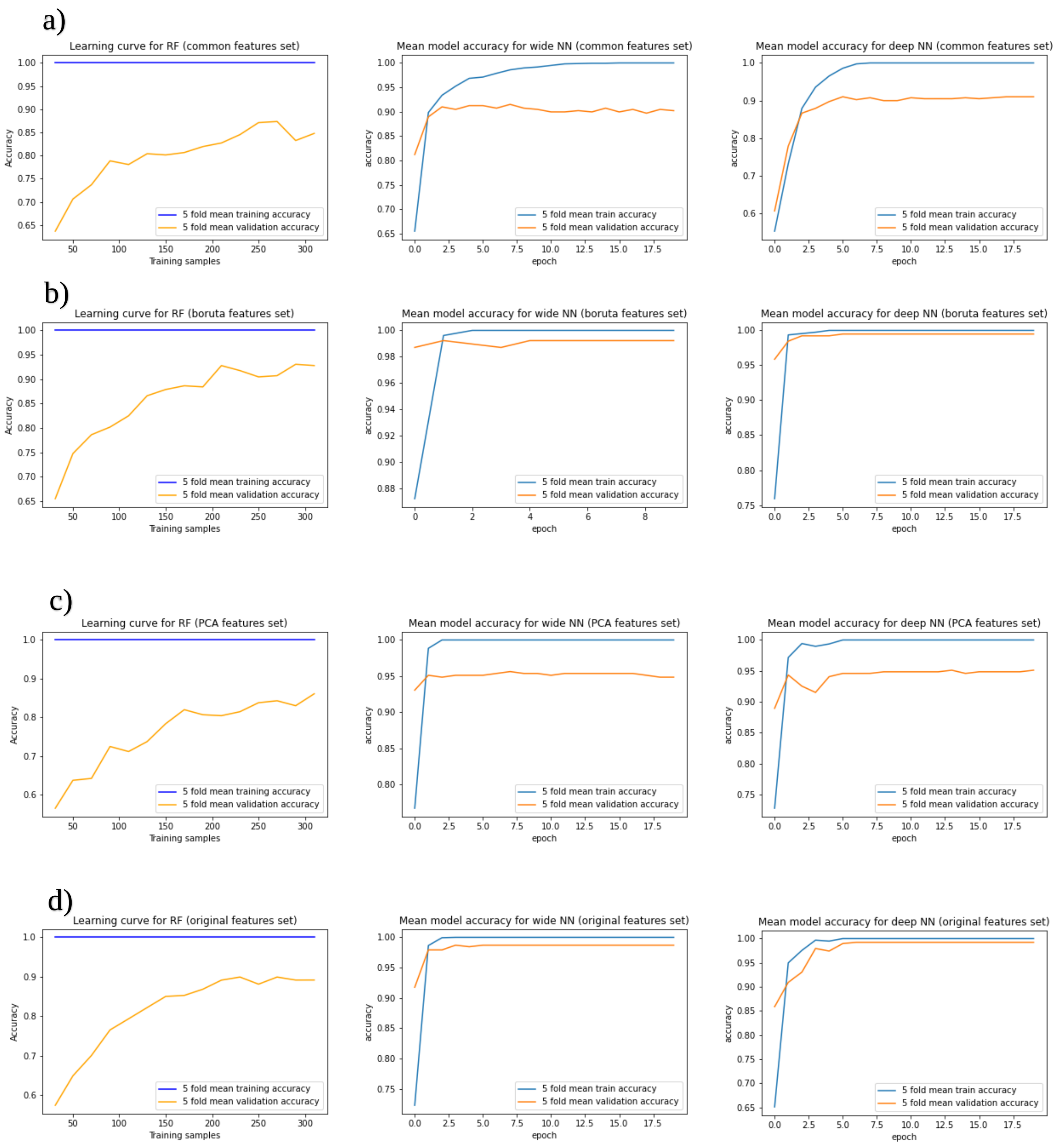

Supplementary Figure 1: accuracy curves for models in (a) EXP1, (b) EXP2, (c) EXP3 and, (d) EXP5

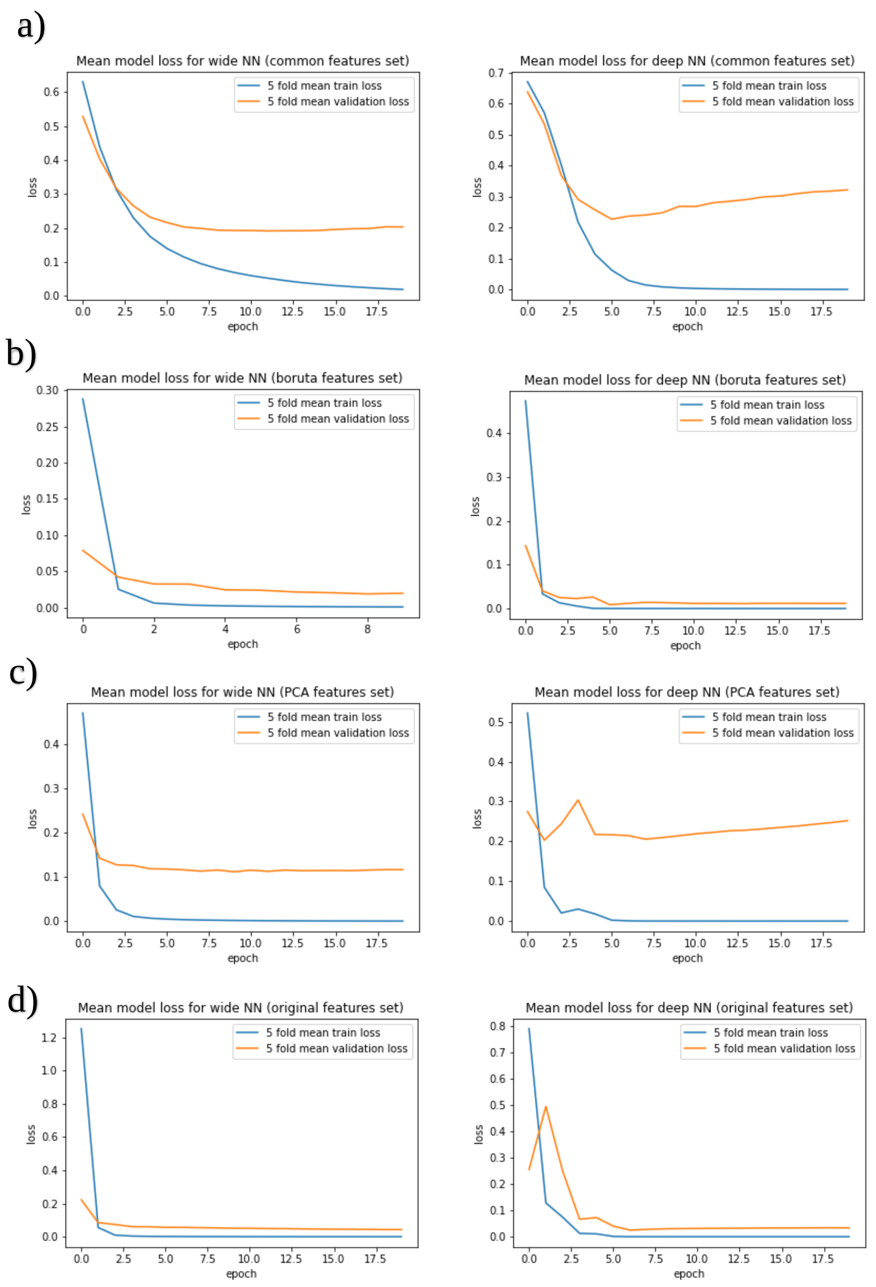

Supplementary Figure 2: loss curves for models in (a) EXP1, (b) EXP2, (c) EXP3 and, (d) EXP5

Supplementary Table 1: Top 50 features selected by Boruta algorithm

rs6116375_CC	rs11768384_GG	rs6585082_TG	rs11706690_CC	rs10491109_AC
rs17365991_GG	rs2075650_AA	rs6505403_TT	rs327079_TT	rs12670401_CC
rs8141950_CC	rs7342676_CC	rs1927605_AA	rs10790928_TT	rs2208322_AA
rs2654986_TC	rs12822144_AA	rs6585082_GG	rs2654986_CC	rs10871809_TC
rs2036109_TT	rs11253696_GG	rs327079_CT	rs4795895_GG	rs1387089_TT
rs12804305_CT	rs4351677_TC	rs10491109_CC	rs6577539_GA	rs1233651_AA

rs10133989_AA	rs4351677_CC	rs2883782_TT	rs11160481_CC	rs3775770_AA
rs17365991_AG	rs12804305_TT	rs4778636_AG	rs1797779_CT	rs3004297_TT
rs8141950_TC	rs3857224_TT	rs7146951_GG	rs7159863_TT	rs1387089_CT
rs2042599_GG	rs4964453_TT	rs1981542_GT	rs4635275_AA	rs4566279_CT

Supplementary Table 2: Paramter setting for ML models in experments 1,2,3,4 and 5

<table>
<tr><td colspan="11">EXP1</td></tr>
<tr><td>RF</td><td colspan="10">(n_estimators = 500, max_features='auto', max_depth=6, criterion='gini'</td></tr>
<tr><td rowspan="2">WNN</td><td colspan="7">No of neurons and activation function in hidden layers</td><td>No of epochs</td><td>Learning rate</td><td>optimizer</td></tr>
<tr><td colspan="7">121 RELU</td><td>15</td><td>0.001</td><td>Adam</td></tr>
<tr><td>DNN</td><td>60 RELU</td><td colspan="2">40 RELU</td><td colspan="2">30 RELU</td><td colspan="2">20 RELU</td><td>15</td><td>0.001</td><td>Adam</td></tr>
<tr><td colspan="11">EXP2</td></tr>
<tr><td>RF</td><td colspan="10">n_estimators = 500, max_features='sqrt', max_depth=7, criterion='gini'</td></tr>
<tr><td rowspan="2">WNN</td><td colspan="7">No of neurons and activation function in hidden layers</td><td>No of epochs</td><td>Learning rate</td><td>optimizer</td></tr>
<tr><td colspan="7">747 RELU</td><td>15</td><td>0.001</td><td>Adam</td></tr>
<tr><td>DNN</td><td>373 RELU</td><td colspan="2">249 RELU</td><td colspan="2">186 RELU</td><td colspan="2">124 RELU</td><td>15</td><td>0.001</td><td>Adam</td></tr>
<tr><td colspan="11">EXP3</td></tr>
<tr><td>RF</td><td colspan="10">n_estimators = 500, max_features='sqrt', max_depth=8, criterion='entropy'</td></tr>
<tr><td rowspan="2">WNN</td><td colspan="7">No of neurons and activation function in hidden layers</td><td>No of epochs</td><td>Learning rate</td><td>optimizer</td></tr>
<tr><td colspan="7">747 RELU</td><td>15</td><td>0.001</td><td>Adam</td></tr>
<tr><td>DNN</td><td>373 RELU</td><td colspan="2">249 RELU</td><td colspan="2">186 RELU</td><td colspan="2">124 RELU</td><td>15</td><td>249 RELU</td><td>186 RELU</td></tr>
<tr><td colspan="11">EXP4</td></tr>
<tr><td></td><td></td><td colspan="6">No of neurons and activation function in hidden layers</td><td>No of epochs</td><td>Learning rate</td><td>optimizer</td></tr>
<tr><td rowspan="2">WDNN</td><td>Wide</td><td colspan="6">100 RELU</td><td rowspan="2">15</td><td rowspan="2">0.001</td><td rowspan="2">Adam</td></tr>
<tr><td>Deep</td><td>2500 RELU</td><td>2000 RELU</td><td>1000 RELU</td><td colspan="2">500 RELU</td><td>100 RELU</td></tr>
<tr><td colspan="11">EXP5</td></tr>
<tr><td>RF</td><td colspan="10">n_estimators = 600, max_features='sqrt', max_depth=7, criterion='entropy'</td></tr>
<tr><td rowspan="2">WNN</td><td colspan="7">No of neurons and activation function in hidden layers</td><td>No of epochs</td><td>Learning rate</td><td>optimizer</td></tr>
<tr><td colspan="7"></td><td></td><td></td><td></td></tr>
<tr><td>DNN</td><td>1497 RELU</td><td colspan="2">998 RELU</td><td colspan="2">748 RELU</td><td colspan="2">499 RELU</td><td>15</td><td>0.001</td><td>Adam</td></tr>
</table>

Supplementary Table 3: Rules extracted from best tree of RF model

if (rs705904_CC > 0.5) and (rs4953672_CC <= 0.5) and (rs799447_GG > 0.5) and (rs701880_CC <= 0.5) then class: Control (proba: 100.0%) | based on 20 samples

if (rs705904_CC <= 0.5) and (rs2075650_AA > 0.5) and (rs1789250_AA <= 0.5) and (rs939720_CC <= 0.5) and (rs268909_TT <= 0.5) and (rs7342676_CC <= 0.5) and (rs1479884_GG <= 0.5) then class: Control (proba: 100.0%) | based on 17 samples

if (rs705904_CC <= 0.5) and (rs2075650_AA <= 0.5) and (rs871049_CC > 0.5) and (rs2577322_TT <= 0.5) and (rs8000805_GG <= 0.5) then class: Case (proba: 100.0%) | based on 14 samples

if (rs705904_CC <= 0.5) and (rs2075650_AA > 0.5) and (rs1789250_AA <= 0.5) and (rs939720_CC <= 0.5) and (rs268909_TT > 0.5) and (rs793291_AA <= 0.5) and (rs7342676_CC <= 0.5) and (rs11922179_AA > 0.5) and (rs628482_GG <= 0.5) and (rs2577322_CC <= 0.5) and (rs1495813_CC <= 0.5) then class: Control (proba: 100.0%) | based on 10 samples

if (rs705904_CC <= 0.5) and (rs2075650_AA > 0.5) and (rs1789250_AA <= 0.5) and (rs939720_CC <= 0.5) and (rs268909_TT > 0.5) and (rs793291_AA <= 0.5) and (rs7342676_CC <= 0.5) and (rs11922179_AA > 0.5) and (rs628482_GG <= 0.5) and (rs2577322_CC <= 0.5) and (rs1495813_CC > 0.5) and (rs11680332_GG <= 0.5) and (rs9296691_TC <= 0.5) and (rs9515168_GT <= 0.5) then class: Control (proba: 100.0%) | based on 9 samples

if (rs705904_CC <= 0.5) and (rs2075650_AA <= 0.5) and (rs871049_CC <= 0.5) and (rs16864809_TT <= 0.5) and (rs1328179_TT <= 0.5) and (rs6116375_CC <= 0.5) and (rs4837137_AA <= 0.5) and (rs3771389_CT <= 0.5) then class: Case (proba: 100.0%) | based on 8 samples

if (rs705904_CC <= 0.5) and (rs2075650_AA <= 0.5) and (rs871049_CC <= 0.5) and (rs16864809_TT <= 0.5) and (rs1328179_TT > 0.5) then class: Case (proba: 100.0%) | based on 6 samples

if (rs705904_CC <= 0.5) and (rs2075650_AA <= 0.5) and (rs871049_CC <= 0.5) and (rs16864809_TT <= 0.5) and (rs1328179_TT <= 0.5) and (rs6116375_CC > 0.5) then class: Control (proba: 100.0%) | based on 6 samples

if (rs705904_CC <= 0.5) and (rs2075650_AA > 0.5) and (rs1789250_AA > 0.5) and (rs871049_CC > 0.5) then class: Case (proba: 100.0%) | based on 5 samples

if (rs705904_CC <= 0.5) and (rs2075650_AA <= 0.5) and (rs871049_CC <= 0.5) and (rs16864809_TT <= 0.5) and (rs1328179_TT <= 0.5) and (rs6116375_CC <= 0.5) and (rs4837137_AA > 0.5) then class: Control (proba: 100.0%) | based on 4 samples

if (rs705904_CC <= 0.5) and (rs2075650_AA <= 0.5) and (rs871049_CC > 0.5) and (rs2577322_TT <= 0.5) and (rs8000805_GG > 0.5) and (rs799447_GG <= 0.5) then class: Case (proba: 100.0%) | based on 4 samples
